# Preparation, characterization, and performance evaluation of a controlled-release inhibitor-enhanced nitrogen fertilizer

**DOI:** 10.3389/fpls.2026.1901755

**Published:** 2026-08-10

**Authors:** Fengru Gao, Zhizhuang An, Jingwei Wang, Weiwen Qiu, Shiyou Sun, Haize Wang, Yishan Chen, Jingxuan Pan, Yu Dong, Xinyu Zhou, Yangyang Xie, Jinling Lv, Qiang Xiao

**Affiliations:** 1 Institute of Plant Nutrition, Resources and Environment, Beijing Academy of Agriculture and Forestry Sciences, Beijing, China; 2 College of Agronomy, Heilongjiang Bayi Agricultural University, Daqing, China; 3 College of Life Science and Technology, Heilongjiang Bayi Agricultural University, Daqing, China; 4 New Zealand Institute for Bioeconomy Science Limited, Hamilton, New Zealand; 5 Institute of Agricultural Resources and Environment, Hebei Academy of Agriculture and Forestry Sciences, Shijiazhuang, China; 6 Institute of Plant Nutrition, Resources and Environment, Henan Academy of Agricultural Sciences, Zhengzhou, China

**Keywords:** ^15^N tracing, 3,4-dimethylpyrazole phosphate (DMPP), controlled-release inhibitor-enhanced nitrogen fertilizer, nitrification inhibition, nitrogen use efficiency

## Abstract

Improving nitrogen fertilizer use efficiency while reducing nitrogen losses is a major challenge for sustainable crop production. In this study, a controlled-release inhibitor-enhanced nitrogen fertilizer (CYRF) was developed by integrating a polyurethane controlled-release membrane with a 3,4-dimethylpyrazole phosphate (DMPP) nitrification-inhibitor layer. Its structural characteristics, nutrient release behavior, and nitrogen-regulating performance were evaluated using scanning electron microscopy, energy-dispersive X-ray spectroscopy, static water incubation, soil incubation at different temperatures and field nitrogen-15 (¹⁵N) tracing. Morphological and elemental analyses confirmed that CYRF possessed a distinct multilayer structure consisting of an outer controlled-release membrane and an inner DMPP-containing inhibitor layer, providing a structural basis for the coordinated release of urea nitrogen and DMPP. Under static water incubation at 25 °C, the cumulative release of urea nitrogen increased from 0.40% at 7 d to 87.50% at 98 d, while DMPP release increased from 0.70% to 79.00% over the same period. DMPP was released slightly earlier than urea nitrogen during the 21–49 d incubation period, and their cumulative release rates were strongly positively correlated (r = 0.975, P < 0.001). Soil incubation showed that CYRF consistently enhanced ammonium nitrogen retention while suppressing nitrate nitrogen accumulation across different temperatures. Compared with the controlled-release fertilizer (CRF), CYRF increased ammonium nitrogen retention by 37.3% and reduced nitrate nitrogen accumulation by 16.4% at 15 °C. At 25 °C, CYRF reduced nitrate nitrogen accumulation by 97.0–98.7% during the 50–90 d incubation period, while at 35 °C it decreased nitrate nitrogen accumulation by 42.0%. Field ¹⁵N tracing further showed that, compared with CRF(^15^N), CYRF(^15^N) significantly increased ^15^N utilization rates by 14.60 and 5.30 percentage points and reduced nitrogen loss rates by 11.45 and 11.11 percentage points in winter wheat and summer maize, respectively. Overall, CYRF effectively synchronized controlled nitrogen release with nitrification inhibition, providing a promising strategy for improving nitrogen use efficiency and reducing nitrogen losses in agricultural production.

## Graphical Abstract

**Figure:**
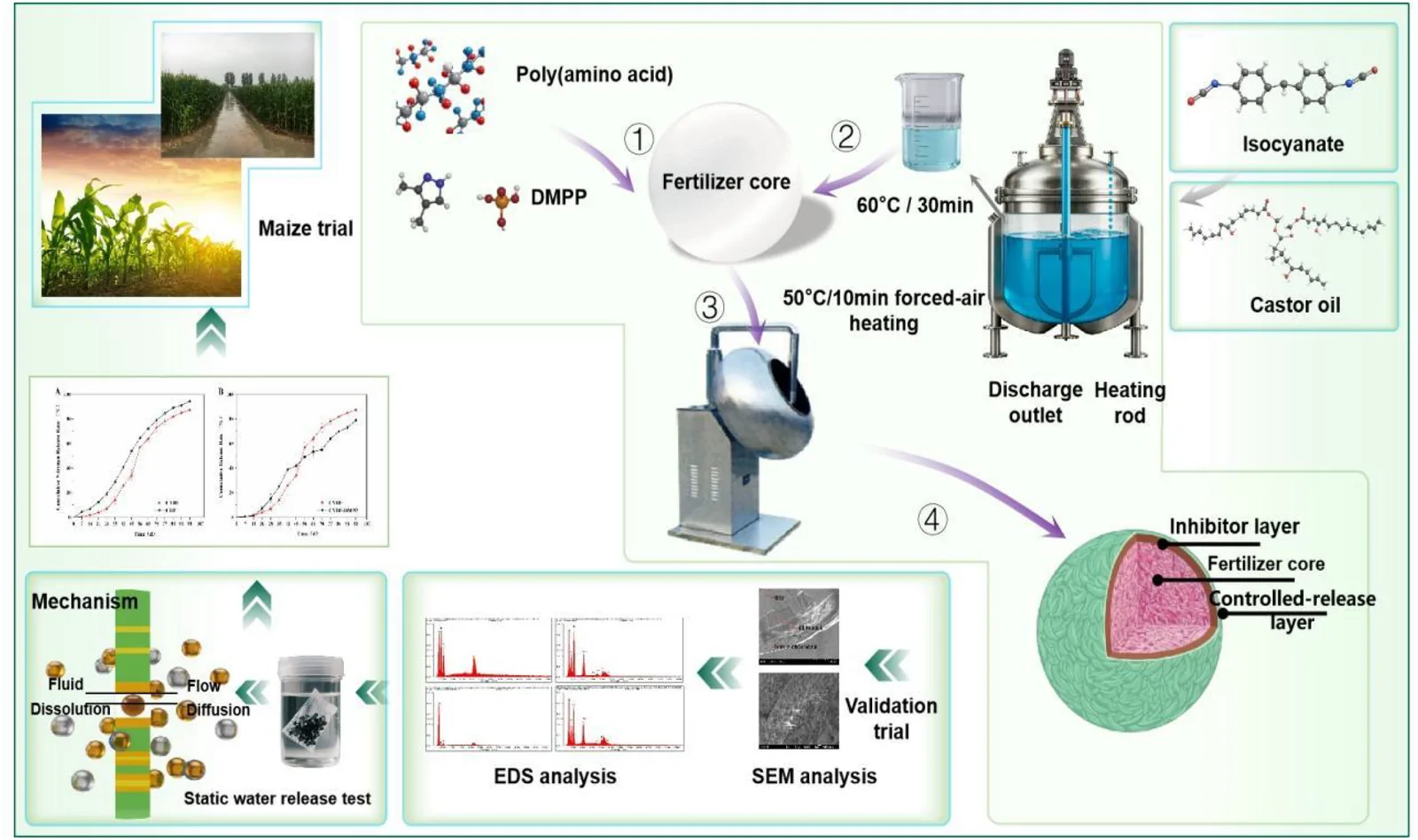
Schematic illustration of the synthesis process of CYRF.

## 1 Introduction

By 2050, global food production is expected to need a 70% increase to support a population of 9.8 billion. Nitrogen fertilizer has played a major role in sustaining crop productivity, contributing more than 50% to long-term improvements in grain yield (Dimkpa et al., 2020). However, the current crop-season nitrogen use efficiency of fertilizer nitrogen is typically below 50%. Inefficient nitrogen use results not only in resource loss, but also in serious environmental consequences, including greenhouse gas emissions, groundwater contamination, and eutrophication of surface waters (Dimkpa et al., 2020; Zhang et al., 2023). Therefore, improving nitrogen fertilizer use efficiency while reducing nitrogen losses is essential for both food security and environmental protection.

Controlled-release nitrogen fertilizers and nitrification-inhibitor-containing nitrogen fertilizers are two important approaches for improving nitrogen fertilizer use efficiency (Dimkpa et al., 2020; Lawrencia et al., 2021; Zhang et al., 2023). Controlled-release fertilizers regulate nutrient release through physical coating, whereas nitrification inhibitors, such as DMPP, slow the oxidation of ammonium following urea hydrolysis. Nevertheless, the field effectiveness of these technologies is often variable. Some studies have shown that polymer-coated controlled-release urea can increase crop yield and nitrogen use efficiency while reducing NH_3_ and N_2_O emissions (Zhang et al., 2019). In contrast, other studies have reported limited or non-significant benefits (Scheer et al., 2016). Similarly, the effects of inhibitor-containing fertilizers on crop yield, nitrogen uptake, and nitrogen loss depend strongly on soil properties and climatic conditions (Meng et al., 2020; Min et al., 2021; Pan et al., 2016; Martins et al., 2017).

The nutrient release characteristics of controlled-release nitrogen fertilizers are largely determined by the properties of the coating material and the pore structure of the membrane. For porous membrane materials, such as polyolefin and polyurethane, nitrogen release is affected by porosity, pore size, tortuosity, water penetration, and diffusion resistance (Lawrencia et al., 2021; Yang et al., 2018; Wei et al., 2017; Wang et al., 2022; Kusters et al., 2023). Although DMPP has considerable potential as a nitrification inhibitor, its performance is sensitive to environmental factors, including temperature, soil moisture, soil type, organic matter content, pH, and other site-specific conditions (Kou et al., 2015; Wu et al., 2017; Guardia et al., 2018; Mariano et al., 2019; Abalos et al., 2017; Yu et al., 2014; Norton and Ouyang, 2019; Guan et al., 2024). These limitations indicate that each fertilizer type has distinct constraints. Controlled-release nitrogen fertilizers can regulate the timing and rate of nitrogen release, but they do not directly control nitrogen transformation once the released nitrogen enters the soil. In contrast, inhibitor-containing fertilizers can delay nitrogen transformation, but because the inhibitor and urea are generally applied together at one time, their effective duration may be short and their performance may vary. Theoretically, combining these two technologies could provide more comprehensive nitrogen regulation: the controlled-release membrane could regulate urea nitrogen release, while the DMPP layer could control post-release nitrification.

Recent studies have further shown that the spatial placement of inhibitors within coated fertilizers can influence nitrogen loss reduction and fertilizer enhancement efficiency. For example, placing the inhibitor between the urea core and the coating layer was more effective in reducing nitrogen losses than uniformly distributing the inhibitor within the coating layer (Qi et al., 2021). In addition, inhibitor-amended coated urea improved nitrogen use efficiency and fertilizer-N allocation in a soil-maize system (Ba et al., 2021). These findings support the feasibility of integrating nitrification inhibitors with coated fertilizer technologies. However, the CYRF product developed in the present study differs from previous products in both formulation and preparation process. In this study, a DMPP-containing controlled-release, inhibitor-enhanced nitrogen fertilizer was developed using liquid-phase suspension, rapid-drying coating, and *in situ* film-forming technologies (Xiao et al., 2021).This study aims to address the following objectives: (1) to characterize the synergistic release patterns of inhibitory materials and nitrogen by analysing membrane material properties and simulating the dissolution process; (2) to determine the characteristics of nitrogen release and transformation under the combined regulation of controlled release and inhibition using incubation experiments; and (3) to evaluate nitrogen use, nitrogen loss, and crop yield responses under the synergistic regulation of controlled release and inhibition using a ¹^5^N tracer experiment.

## 2 Materials and methods

### 2.1 Materials

Large-granular urea with a particle size of 2.5–3.5 mm was selected as the fertilizer core (U, 46% N). The nitrification inhibitor used in this study was 3, 4-dimethylpyrazole phosphate (DMPP). Sodium polyaspartate (PASD) served as the carrier and dispersing medium to facilitate DMPP loading onto the urea surface. No urease inhibitor, such as NBPT, was incorporated into any of the fertilizer formulations. Polyurethane was used as the coating material, with isocyanate (YQSZ) as the principal component. Paraffin wax (SL), butanediol (DEC), and castor oil (BMY) were used as auxiliary additives. In the soil incubation experiment, the stabilized fertilizer treatment (CN) represented DMPP-coated urea without an external polyurethane controlled-release membrane. This treatment was prepared using the same DMPP-loading method as YU1 and was included to distinguish the effect of nitrification inhibition from that of controlled release. CRF represented polyurethane-coated urea without DMPP, whereas CYRF represented DMPP-coated urea that was subsequently coated with the polyurethane controlled-release membrane.

### 2.2 Fertilizer preparation

#### 2.2.1 Preparation of DMPP-coated urea

DMPP-coated urea, referred to as YU and used as the CN treatment in the soil incubation experiment, was prepared using a rotary drum coater (WKY-400, China). The coating conditions were set as follows: inlet air temperature, 50 °C; compressed air pressure, 0.3 Pa; and nozzle atomization pressure, 0.2 Pa. Urea granules (1.0 kg) were added to the rotary drum coater and preheated at 50 ± 2 °C for 10 min. For YU1 and YU2, 9.2 g and 2.3 g of DMPP were separately dissolved or evenly dispersed in 46.0 g and 11.5 g of PASD-containing carrier solution, respectively. After complete mixing, each DMPP suspension was sprayed onto the surface of the urea granules. The resulting coated granules were dried to obtain YU1 and YU2. The inhibitor layer contained only DMPP, and no NBPT was added. YU1 was used as the CN treatment in the soil incubation experiment, while YU1 and YU2 were further coated to produce CYRF1 and CYRF2, respectively.

#### 2.2.2 Preparation of controlled-release inhibitor-enhanced nitrogen fertilizer

Controlled-release inhibitor-enhanced nitrogen fertilizer (CYRF) was produced using an *in situ* reactive film-forming method. Briefly, 0.13 g of paraffin wax, 0.17 g of butanediol, and 1.2 g of castor oil were placed in a beaker and heated until fully melted to form component A. Component B consisted of 1.5 g of isocyanate. Components A and B were then sprayed sequentially onto the surfaces of YU1 and YU2 under rotary spraying conditions with forced air at 50 °C. This coating process was repeated three times. After drying, smooth, continuous, and uniform controlled-release inhibitor-enhanced nitrogen fertilizers were obtained and designated as CYRF1 and CYRF2. The coating materials accounted for approximately 2.7% and 2.9% of the urea mass, respectively, and the corresponding nitrogen contents were approximately 43.8% and 44.3%. According to the national standard, the release period was approximately 50–60 days. CYRF1 was used in the laboratory incubation experiment, whereas CYRF2 was used in the field micro-plot experiment. Therefore, in this study, CYRF refers to a DMPP-containing controlled-release fertilizer rather than a product combining both urease and nitrification-inhibitors.

#### 2.2.3 Morphological, cross-sectional structural, and energy-dispersive spectroscopic analysis of coated fertilizers

The surface morphology, cross-sectional membrane structure, and film-forming characteristics of the coated fertilizers were examined using an S-450 scanning electron microscope (SEM). The elemental composition and energy-dispersive spectral characteristics of the fertilizers and coating materials were analyzed using energy-dispersive X-ray spectroscopy (EDS).

#### 2.2.4 Determination of urea nitrogen

A 5.0 g sample of coated fertilizer was transferred into a 100 mL beaker and soaked in 60 mL of distilled water. The fertilizer granules were crushed using a glass rod and allowed to dissolve completely. The solution was then filtered and quantitatively transferred into a 1000 mL volumetric flask. The beaker, filter paper, and remaining coating residues were rinsed repeatedly with distilled water, and all rinsing solutions were collected in the same volumetric flask. The volume was adjusted to the calibration mark with distilled water to prepare the stock solution. The stock solution was then diluted 100-fold, and urea nitrogen content was measured using an AutoAnalyzer 3 continuous-flow analyzer (Bran+Luebbe).

#### 2.2.5 Determination of DMPP in the inhibitor material

The DMPP content in the inhibitor material was determined following the method specified in the Chinese agricultural industry standard NY/T 3423-2019.

### 2.3 Laboratory incubation

#### 2.3.1 Static water incubation experiment

A static water incubation experiment was carried out at 25 °C to determine the basic release behaviour of the controlled-release fertilizer and the controlled-release inhibitor-enhanced nitrogen fertilizer. Controlled-release fertilizer (CRF) and controlled-release inhibitor-enhanced nitrogen fertilizer (CYRF) were selected as the test materials. The CRF treatment was used to describe the release pattern of urea nitrogen from the controlled-release coating, while the CYRF treatment was used to assess the release characteristics of both urea nitrogen and the nitrification inhibitor DMPP. The results from this experiment provided provided a foundation for interpreting differences in ammonium nitrogen retention and nitrate nitrogen accumulation observed in subsequent incubation experiments conducted at different temperatures.

For each treatment, 10 g of uniformly sieved fertilizer was weighed and placed in a nylon mesh bag, which was then transferred into a plastic bottle. Deionized water, 250 mL, was added to fully immerse the fertilizer granules. The bottles were incubated statically in the dark at 25 °C. Extracts were collected after 7, 14, 21, 28, 35, 42, 49, 56, 63, 70, 77, 84, 91, and 98 days of incubation. At each sampling time, the entire solution was removed and replaced with the same volume of fresh deionized water. Urea nitrogen and DMPP concentrations in the extracts were measured, and cumulative release rates were calculated based on the accumulated release at each sampling point. Urea nitrogen and DMPP were determined according to the methods described in Sections 1.2.4 and 1.2.5, respectively. Each treatment was conducted in triplicate.

#### 2.3.2 Soil incubation experiment

Static water incubation mainly reflects the dissolution and diffusion behaviour of fertilizer granules in water. However, after fertilizer is applied to soil, nitrogen release and transformation are also influenced by urease activity, nitrifying microorganisms, soil adsorption and fixation, temperature, and other soil processes. Therefore, after clarifying the release patterns of urea nitrogen and DMPP from CYRF, soil incubation experiments were conducted at 15 °C, 25 °C, and 35 °C to evaluate the effects of CYRF on ammonium nitrogen retention and nitrate nitrogen accumulation under soil conditions.

A typical loamy fluvo-aquic soil was collected from a winter wheat-growing area in the North China Plain and used as the incubation medium. Stones and plant residues were manually removed. and the soil was passed through a 2 mm sieve before being air-dried in a cool and well-ventilated place. The basic physicochemical properties of the soil were as follows: pH 7.9 at a soil-to-water ratio of 1:2.5, organic matter content 38.6 g/kg, available phosphorus 3.8 mg/kg, available potassium 114 mg/kg, and total nitrogen 0.4 g/kg. Overall, the soil was slightly alkaline and had a moderate fertility level.

For the incubation experiment, 300 g of air-dried soil passed through a 2.00 mm sieve was placed in plastic boxes. Five treatments were established in triplicate: no fertilizer (CK), conventional urea (U), DMPP-stabilized urea without an outer controlled-release membrane (CN), controlled-release urea without DMPP (CRF), and DMPP-containing controlled-release inhibitor-enhanced nitrogen fertilizer (CYRF). Nitrogen fertilizers were applied at 0.8 g N/kg soil and thoroughly mixed with the soil. Soil moisture was adjusted to 80% of field water-holding capacity, and samples were incubated in the dark at 15, 25, and 35 °C. Moisture was maintained by regular weighing and water addition. Destructive sampling was conducted at 3, 7, 14, 21, 35, 50, 65, and 90 d, after which the soil was homogenized for ammonium and nitrate nitrogen determination.

### 2.4 Evaluation of nitrogen regulation characteristics using a field nitrogen-15 tracing experiment

The field experiment was conducted in Dahe Village, Dahe Town, Luquan, Shijiazhuang, Hebei Province, which is located in the semi-humid plain region of the Huang-Huai-Hai area and has a climate with four distinct seasons. The tested soil was loamy fluvo-aquic soil with a moderate fertility level. The basic properties of the 0–20 cm soil layer were as follows: organic matter, 16.29 g/kg; total nitrogen, 0.70 g/kg; total phosphorus, 0.06 g/kg; total potassium, 1.82%; available phosphorus, 5.40 mg/kg; available potassium, 62.02 mg/kg; and pH, 8.34.

The summer maize and winter wheat cultivars used in the field experiment were Zhengdan 958 and Lunxuan 987, respectively. Seven fertilization treatments were established for each crop, as shown in **Tables 1** and **2**. The ¹^5^N-labeling patterns differed among treatments: in some treatments, only the target nitrogen fertilizer component was ¹^5^N-labeled, whereas in the remaining treatments, all nitrogen fertilizer components were ¹^5^N-labeled. Treatments in which only the controlled-release fertilizer component was ¹^5^N-labeled were denoted as CRF(¹^5^N), whereas those in which only the controlled-release inhibitor-enhanced nitrogen fertilizer component was ¹^5^N-labeled were denoted as CYRF(¹^5^N). The ¹^5^N-labeled fertilizer had an abundance of 10.2% and was purchased from the Shanghai Research Institute of Chemical Industry. Each treatment had three replicates. Cylindrical micro-plots of 0.126 m^2^ containing 15 plants were used for winter wheat, whereas square micro-plots of 0.25 m^2^ containing two plants were used for summer maize. The micro-plots were separated by PVC boards buried to a depth of 0.6 m to prevent lateral seepage and were randomly arranged.

**Table 1:** Fertilization treatments for winter wheat with nitrogen-15 (¹^5^N) tracing.

No	Treatment	Fertilizer type	N reduction	Basal N application kg·hm^-^²	Topdressing N application kg·hm^-^²
T1	Conventional basal fertilization + optimized topdressing (^15^N)	Conventional urea + ^15^N-labeled urea	30% reduction in topdressing N	142.5	99.75
T2	Conventional basal fertilization (^15^N) + optimized topdressing (^15^N)	^15^N-labeled urea + ^15^N-labeled urea	30% reduction in topdressing N	142.5	99.75
T3	Optimized basal fertilization (^15^N) + optimized topdressing (^15^N)	^15^N-labeled urea + ^15^N-labeled urea	30% reduction in total N	99.75	99.75
T4	One-time basal application, optimized treatment	^15^N-labeled controlled-release urea (60 d) + conventional urea (4:6)	30% reduction in total N	199.5	
T5	One-time basal application, optimized treatment	^15^N-labeled controlled-release urea (60 d) + ^15^N-labeled conventional urea (4:6)	30% reduction in total N	199.5	
T6	One-time basal application, optimized treatment	^15^N-labeled controlled-release inhibitor urea (60 d) + conventional urea (4:6)	30% reduction in total N	199.5	
T7	One-time basal application, optimized treatment	^15^N-labeled controlled-release inhibitor urea (60 d) + ^15^N-labeled conventional urea (4:6)	30% reduction in total N	199.5	

**Table 2:** Fertilization treatments for summer maize with nitrogen-15 (¹^5^N) tracing.

No.	Treatment	Fertilizer type	N reduction	Basal N application kg·hm^-^²	Topdressing N application kg·hm^-^²
T1	Conventional basal fertilization + optimized topdressing (¹^5^N)	Conventional urea + ¹^5^N-labeled urea	30% reduction in topdressing N	112.5	78.75
T2	Conventional basal fertilization (¹^5^N) + optimized topdressing (¹^5^N)	¹^5^N-labeled urea + ¹^5^N-labeled urea	30% reduction in topdressing N	112.5	78.75
T3	Optimized basal fertilization (¹^5^N) + optimized topdressing (¹^5^N)	¹^5^N-labeled urea + ¹^5^N-labeled urea	30% reduction in total N	78.75	78.75
T4	One-time basal application, optimized treatment 3	^15^N-labeled controlled-release urea (60 d) + conventional urea (3:7)	30% reduction in total N	157.5	
T5	One-time basal application, optimized treatment 3	¹^5^N-labeled controlled-release urea (60 d) + ¹^5^N-labeled conventional urea (3:7)	30% reduction in total N	157.5	
T6	One-time basal application, optimized treatment 1	¹^5^N-labeled controlled-release inhibitor urea (60 d) + conventional urea (3:7)	30% reduction in total N	157.5	
T7	One-time basal application, optimized treatment 1	¹^5^N-labeled controlled-release inhibitor urea (60 d) + ¹^5^N-labeled conventional urea (3:7)	30% reduction in total N	157.5	

Summer maize was sown on June 26, 2024, and topdressing was applied at the small trumpet stage. Winter wheat was sown on October 15, 2024, and topdressing was applied at the jointing stage. The ¹^5^N tracing experiments for the two crops were conducted independently in separate micro-plots. Labeled micro-plots were not reused across crop seasons, and natural-abundance samples from the corresponding unlabeled treatments were used as background values for Ndff calculation. Therefore, ¹^5^N utilization, residual, and loss rates were calculated independently for each crop season without considering cross-seasonal carry-over.

Phosphorus and potassium fertilizers were applied once as basal fertilizers according to local practices. The application rates of P_2_O_5_ and K_2_O were 75 and 90 kg·hm^-2^ for summer maize and 90 and 60 kg·hm^-2^ for winter wheat, respectively. All treatments received equal phosphorus, potassium, and irrigation inputs. Irrigation, weed control, and other field management followed local practices, with micro-sprinkler irrigation used.

### 2.5 Sampling, data calculation, and statistical analysis

At harvest, yield was determined by sampling three complete rows of maize plants from each plot, while wheat yield was measured from a harvested area of 2 m². Crop yield was recorded after natural air-drying. At the same time, 3–10 plants were collected from each plot for the determination of plant nitrogen content. After crop harvest, soil samples were taken from the 0–200 cm soil profile at 20 cm intervals using a soil auger, and soil nitrogen content was subsequently measured.

Data processing and correlation analyses were performed using Microsoft Office Excel 2010, and statistical analyses using SPSS 17.0. Data are presented as means ± SD of three replicates. Treatment effects were analysed by one-way ANOVA followed by the LSD test at P < 0.05, with different lowercase letters indicating significant differences. Pearson correlation analysis assessed the relationship between cumulative urea nitrogen and DMPP release.

The ¹^5^N abundance of total nitrogen in soil and plant samples was determined using a stable isotope ratio mass spectrometer (vario PYRO cube, Elementar, Germany).

The percentage and amount of nitrogen derived from fertilizer in the samples were calculated as follows:

Ndff% = (¹^5^Nsample − ¹^5^N_0_)/(¹^5^Nfertilizer − ¹^5^N_0_).

Ndff = Nsample × Ndff%.

where Ndff% is the proportion of sample nitrogen derived from ¹^5^N-labeled urea; Ndff is the corresponding fertilizer-derived nitrogen amount; ¹^5^Nsample, ¹^5^N_0_, and ¹^5^Nfertilizer are the ¹^5^N abundances of the labeled sample, unlabeled control, and fertilizer, respectively; and Nsample is the sample nitrogen content expressed as kg N ha^-^¹.

## 3 Results and analysis

### 3.1 Surface morphology and cross-sectional structure of coated fertilizers

**Figure 1:**
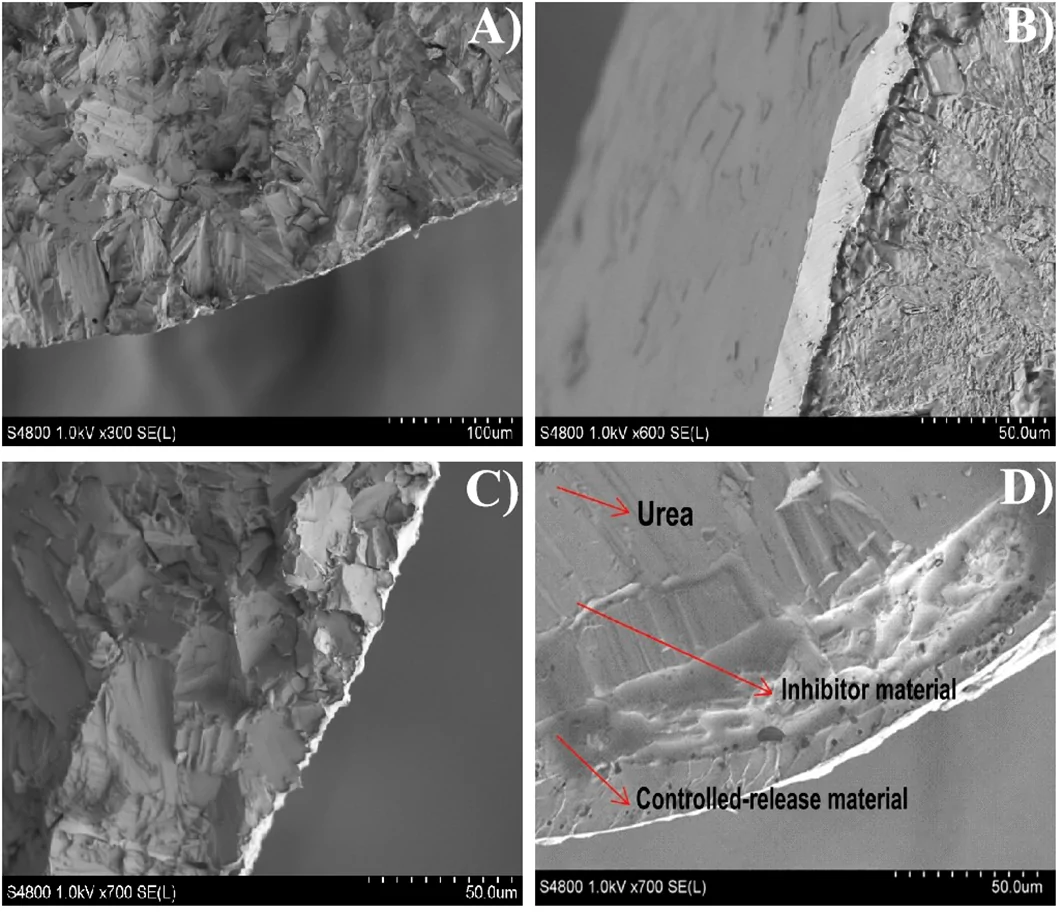
Cross-sectional scanning electron microscope (SEM) images of different fertilizer samples. **(A)** Conventional urea; **(B)** controlled-release fertilizer (CRF) coating; **(C)** inhibitor coating; **(D)** controlled-release inhibitor-enhanced nitrogen fertilizer (CYRF) with an inner inhibitor layer and an outer polyurethane (PU) layer.

Different fertilizer modification approaches produced distinct cross-sectional structures (**Figure 1**). Conventional urea had no coating, whereas CRF had a continuous, compact polyurethane membrane tightly attached to the urea core, with a uniform thickness of approximately 75–110 μm, providing a physical basis for controlling water entry and urea nitrogen diffusion. DMPP-coated urea showed a visible inhibitor layer that followed the uneven core surface and filled depressions, indicating effective DMPP loading; however, without an outer controlled-release membrane, it could dissolve and release rapidly. CYRF exhibited a clear multilayer structure consisting of an inner DMPP-containing inhibitor layer and an outer polyurethane membrane. The closely connected layers formed an integrated coating system in which the outer membrane regulated water penetration and solute diffusion, while the inner layer supplied DMPP, supporting synchronized urea nitrogen and DMPP release.

### 3.2 Energy-dispersive spectroscopic analysis of materials and fertilizers

**Figure 2:**
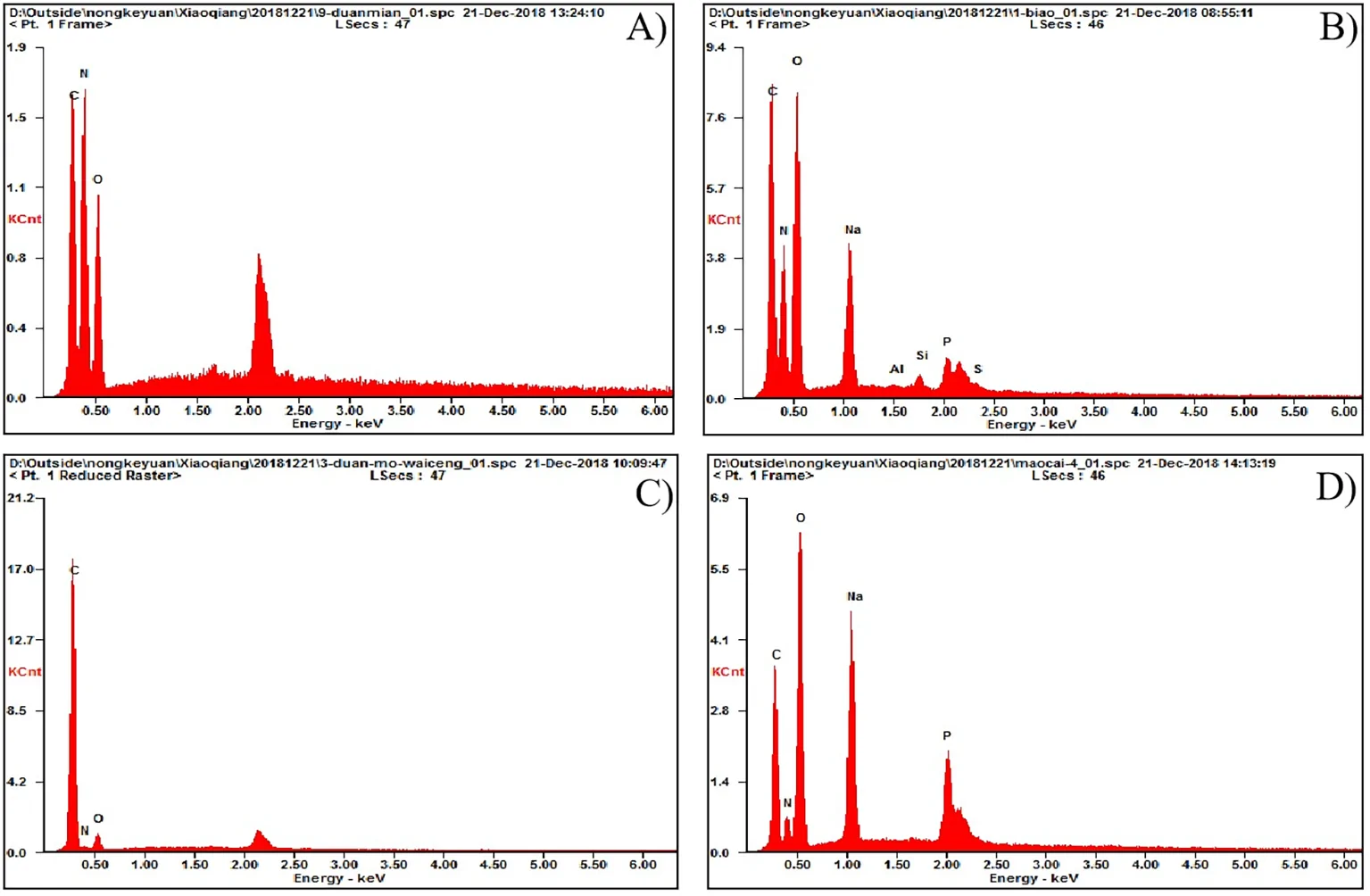
EDS spectra of different fertilizer samples. **(A)** Conventional urea; **(B)** CRF coating; **(C)** inhibitor coating; **(D)** CYRF coating. The presence of Na and P confirms the incorporation of sodium polyaspartate and DMPP in the CYRF coating.

Energy-dispersive spectroscopic (EDS) analysis was conducted to confirm the elemental composition of the coating layers and to verify their successful attachment to the urea granules (**Figure 2**). Conventional urea was mainly composed of carbon, nitrogen, and oxygen, with nitrogen showing the strongest signal (**Figure 2A**).

For DMPP-coated urea, sodium and phosphorus signals were detected (**Figure 2B**), originating from the carrier material and the phosphate group of DMPP, respectively, confirming successful formation of the DMPP-containing inhibitor layer. Because NBPT was not used, sulfur was not considered evidence of urease inhibitor addition.

For CYRF, the outer controlled-release membrane mainly contained carbon, nitrogen, and oxygen, with the strong carbon signal consistent with polyurethane (**Figure 2C**), whereas the inner layer showed distinct sodium and phosphorus signals, confirming the DMPP-containing layer beneath the membrane (**Figure 2D**). These results verified the hierarchical structure integrating nutrient-release regulation and inhibitor delivery within a single granule.

### 3.3 Laboratory static water release experiment

#### 3.3.1 Release characteristics of CYRF under static water incubation at 25 °C

**Figure 3:**
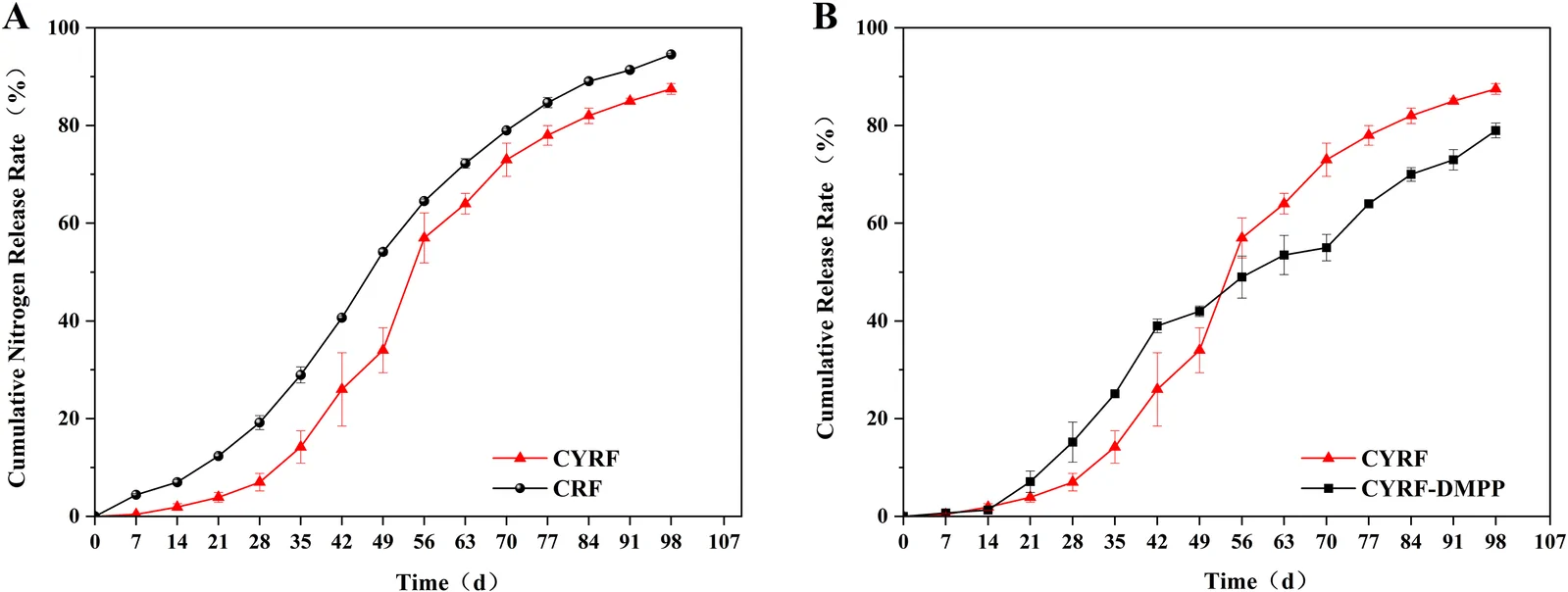
Cumulative release of urea nitrogen and DMPP under static water incubation at 25 °C. **(A)** Urea nitrogen release from CRF and CYRF. **(B)** Urea nitrogen and DMPP release from CYRF.

Under static water incubation at 25 °C, both CRF and CYRF exhibited slow-release characteristics, but urea nitrogen was released more rapidly from CRF (**Figure 3A**). At 7, 28, 49, and 98 d, the cumulative release rates were 4.40%, 19.19%, 54.16%, and 94.54% for CRF, compared with 0.40%, 7.00%, 34.00%, and 87.50% for CYRF, indicating slower early-stage release and a lower risk of burst release from CYRF.

The slower release from CYRF may be attributed to its composite coating structure. In CRF, urea nitrogen release is mainly controlled by the outer controlled-release membrane, which regulates water penetration and nutrient diffusion. In contrast, CYRF contains an additional inhibitor layer beneath the outer membrane. This inner layer may lengthen the diffusion pathway for water entry and solute movement, thereby increasing release resistance and delaying urea nitrogen release.

Comparison of urea nitrogen and DMPP release from CYRF showed that cumulative DMPP release was only 0.70% at 7 d and 1.30% at 14 d, indicating no substantial early-stage release **Figure 3B**. It then increased markedly from 21 d onward, reaching 15.20%, 39.00%, and 79.00% at 28, 42, and 98 d, respectively. DMPP was released relatively earlier than urea nitrogen during the 21–49 d period; at 28 d, its cumulative release rate was 15.20%, higher than that of urea nitrogen at 7.00%, suggesting that DMPP could enter the surrounding environment before the main urea nitrogen release stage and help establish an early nitrification-inhibiting effect.

After 56 days, the cumulative urea nitrogen release rate of CYRF exceeded that of DMPP, indicating a certain temporal coordination between the two release processes. The cumulative release rate of DMPP was extremely significantly and positively correlated with that of urea nitrogen in CYRF (r = 0.975, P < 0.001), indicating showed highly consistent release trends. Overall, CYRF delayed urea nitrogen release through the controlled-release membrane, while allowing DMPP to be released relatively earlier during the early-to-middle stage and continuously released during the later stage, providing pattern provides a basis for the synergistic regulation of “controlled release–inhibited transformation” in subsequent nitrogen regulation processes.

The static water incubation experiment terminated at 98 days because this duration covered both the main release period of the 50–60 day controlled-release fertilizer and the primary observation period of the soil incubation experiment. By 98 days, the cumulative DMPP release rate had reached 79.00%, indicating that DMPP release was still ongoing but had already extended across the main observation stage and was expected to continue slowly beyond this point. Therefore, the time required for complete DMPP release was not directly determined or used as a major evaluation endpoint. Instead, the results demonstrate that CYRF maintained sustained DMPP availability throughout the main urea nitrogen release period, which is important for synchronizing inhibitor availability with crop nitrogen demand and post-release nitrification control.

### 3.4 Nitrogen regulation characteristics of different fertilizer treatments at different temperatures

#### 3.4.1 Nitrogen regulation characteristics of each fertilizer treatment under different temperature conditions

**Figure 4:**
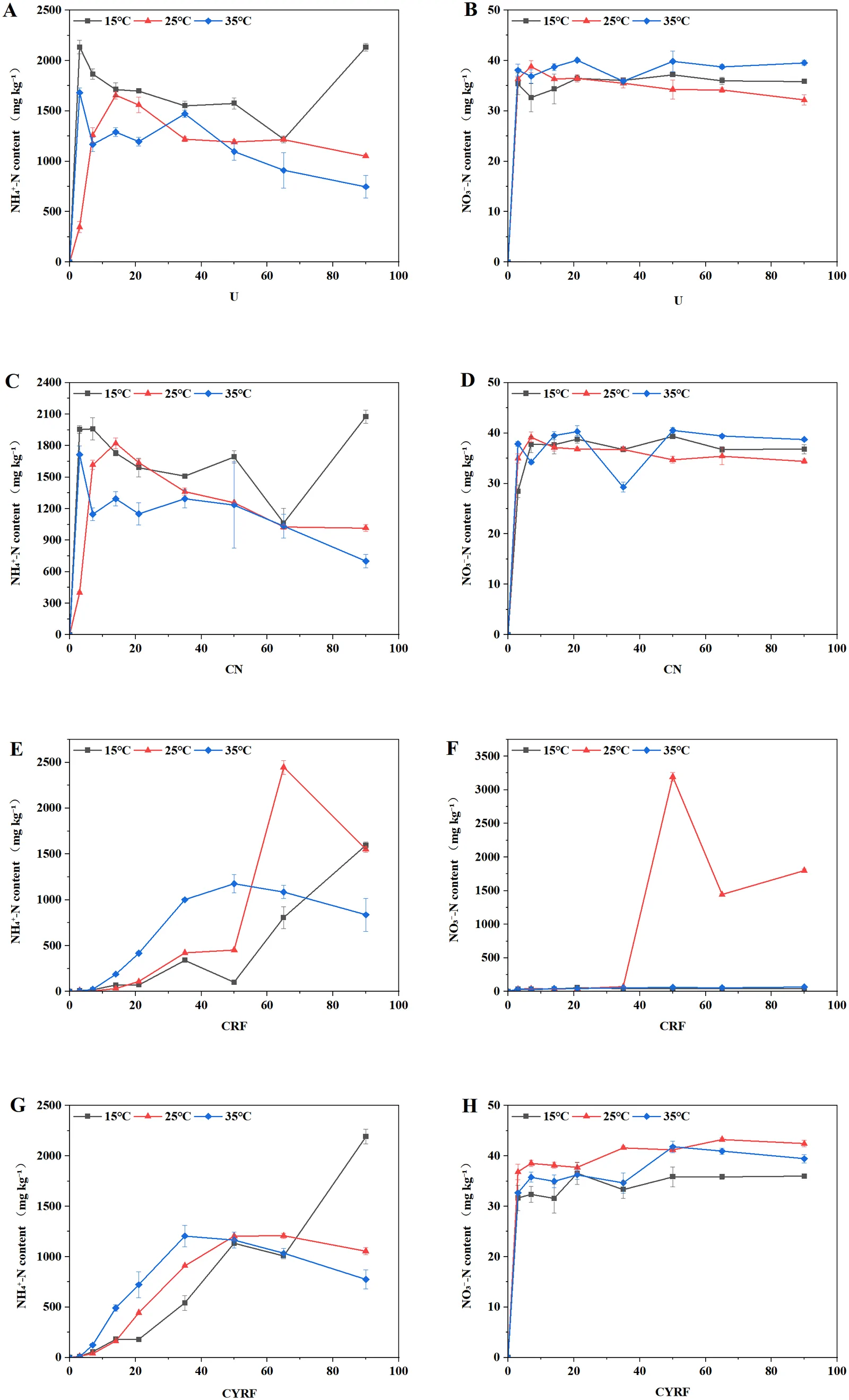
Soil ammonium and nitrate nitrogen dynamics under different treatments and temperatures. **(A–H)** represent changes in soil ammonium nitrogen and nitrate nitrogen under U, CN, CRF, and CYRF treatments, respectively.

The morphological and EDS analyses confirmed that the controlled-release membrane and inhibitor layer were successfully attached to the urea granules, providing a structural foundation for regulating urea nitrogen release and subsequent soil nitrogen transformation. To further evaluate the temperature-dependent regulation of nitrogen release and transformation by CYRF in soil, incubation experiments were conducted at different temperatures to analyse dynamic changes in ammonium nitrogen and nitrate nitrogen for each fertilizer treatment **Figure 4**.

For the U treatment, ammonium nitrogen was rapidly produced during the early incubation stage and then declined to different degrees depending on temperature. Urea nitrogen was hydrolysed by urease and converted into ammonium nitrogen, and this process, together with the subsequent fate of nitrogen, was influenced by soil temperature, moisture, pH, and other environmental factors. At 15 °C, ammonium nitrogen remained relatively stable throughout the incubation period, with almost no decline at 90 d compared with 3 d. This indicates that low temperature slowed ammonia volatilization, nitrification, and other ammonium-consuming processes. At 25 °C, ammonium nitrogen gradually decreased after reaching its maximum value, and the concentration at 90 d was 36.5% lower than the peak value. It was also 1081.39 mg kg^-^¹ lower than that at 15 °C, equivalent to a decrease of approximately 50.7%. By 49 d, it had decreased by 55.5% compared with 3 d, and by the end of incubation it was 1385.28 and 303.89 mg kg^-^¹ lower than the values at 15 °C and 25 °C, respectively, corresponding to reductions of approximately 64.9% and 28.9%. These results suggest that high temperature promoted both ammonia volatilization and nitrification, contributing to the lowest residual ammonium nitrogen content during the later incubation stage.

For nitrate nitrogen, the U treatment showed temperature-dependent differentiation after 35 d, with the order at 90 d being 35 °C > 15 °C > 25 °C. Compared with 25 °C, nitrate nitrogen at 35 °C and 15 °C was higher by 7.39 mg/kg and 3.70 mg/kg, with increases of 23.0% and 11.5%, respectively. The value at 35 °C was 3.69 mg/kg higher than that at 15 °C, corresponding to an increase of approximately 10.3%. This result indicates that nitrate nitrogen content did not simply increase with temperature, but was jointly determined by ammonium nitrogen substrate supply, nitrification intensity, and nitrogen loss. At 15 °C, nitrification was relatively weak, but ammonium nitrogen retention was high. At 35 °C, residual ammonium nitrogen was lower, but nitrification was stronger. At 25 °C, ammonium nitrogen supply was lower than at 15 °C, while nitrification intensity was weaker than at 35 °C, resulting in the lowest final nitrate nitrogen retention in soil.

The ammonium nitrogen dynamics under the CN treatment were generally similar to those under the U treatment, although the stabilizing component delayed ammonium transformation to nitrate to some extent. At 15 °C, ammonium nitrogen remained at a relatively high level and was slightly higher at 90 d than at 3 d, indicating slower ammonium nitrogen consumption under low-temperature conditions. At 25 °C, ammonium nitrogen declined after reaching its peak, with the value at 90 d being 44.2% lower than the peak value. It was also 1059.67 mg kg^-^¹ lower than that at 15 °C during the same period, equivalent to a decrease of approximately 51.0%. At 49 d, it was 59.1% lower than at 3 d, and at the end of incubation it was 1374.94 and 315.27 mg kg^-^¹ lower than the values at 15 °C and 25 °C, respectively, corresponding to decreases of approximately 66.2% and 31.0%. These findings indicate that increasing temperature accelerated the depletion of ammonium nitrogen after its formation, with ammonia volatilization and nitrification jointly reducing residual ammonium nitrogen during the later stage.

For nitrate nitrogen under the CN treatment, no distinct accumulation peak was observed at any of the three temperatures, suggesting that the stabilizing component exerted a nitrification-inhibiting effect. After 50 d, nitrate nitrogen showed temperature-dependent differentiation, generally following the order of 35 °C as the highest, 15 °C as intermediate, and 25 °C as the lowest. At 90 d, nitrate nitrogen concentrations at 35 °C and 15 °C were higher than that at 25 °C by 4.33 and 2.36 mg kg^-^¹, representing increases of approximately 12.6% and 6.9%, respectively. The value at 35 °C was 1.97 mg kg^-^¹ higher than that at 15 °C, equivalent to an increase of approximately 5.4%. These results suggest that nitrate nitrogen dynamics under CN were also not governed by temperature alone, but by the combined effects of ammonium substrate availability, nitrification intensity, and inhibition by the stabilizing component. At 15 °C, more ammonium substrate was retained; at 35 °C, nitrification was stronger; whereas at 25 °C, the system had neither the higher ammonium supply observed at 15 °C nor the stronger nitrification capacity observed at 35 °C, leading to the the lowest nitrate nitrogen content in the later stage.

At 15 °C, ammonium nitrogen mainly accumulated at the later stage of incubation. At 25 °C, the high ammonium nitrogen value appeared at 65 d, while at 35 °C it shifted to 49 d, indicating that increasing temperature accelerated water penetration into the coating, urea nitrogen dissolution, and diffusion through the membrane. At 90 d ammonium nitrogen at 25 °C was 36.5% lower than the peak value. At the end of incubation, ammonium nitrogen at 35 °Cwas approximately 47.6% and 46.1% lower than at 15 °C and 25 °C, respectively.

For nitrate nitrogen, the CRF treatment did not show a simple increasing trend with temperature; instead, marked accumulation occurred at 25 °C. At 90 d, nitrate nitrogen at 25 °C was approximately 41.8 and 26.5 times that at 15 °C and 35 °C, respectively, indicating that ammonium nitrogen released from the coating was more readily transformed and accumulated as nitrate nitrogen.Ammonia volatilization, microbial utilization, or other nitrogen loss pathways may also have been enhanced thereby limiting nitrate nitrogen accumulation. The final nitrate nitrogen content at 35 °C was only approximately 3.8% of that at 25 °C, although it was still 1.58 times that at 15 °C. CRF could delay fertilizer nitrogen release, but its ability to regulate the transformation of ammonium nitrogen into nitrate nitrogen was limited.

At 15 °C, ammonium nitrogen mainly accumulated at the later stage of incubation. At 25 °C, ammonium nitrogen at 90 d still retained 87.4% of the peak value, decreasing by only 12.6%. At 35 °C, the high ammonium nitrogen value appeared earlier than at 25 °C, and only 64.3% of the peak value was retained at the end of incubation, corresponding to a decrease of 35.7%. At the end of incubation, ammonium nitrogen at 35 °C was 1415.89 mg/kg and 280.23 mg/kg lower than those at 15 °C and 25 °C, respectively, approximately 64.6% and 26.6%. The value at 25 °C was also 1135.66 mg/kg lower than that at 15 °C, corresponding to a decrease of approximately 51.8%.

For nitrate nitrogen, CYRF showed no sharp increase at any temperature, and variation was much smaller than that of ammonium nitrogen. Nitrate nitrogen fluctuated within 4.44 mg/kg at 15 °C, increased by 15.2% at 25 °C, and decreased by 5.6% after 42 d at 35 °C. At the end of incubation, the value at 25 °C was only 3.00 mg/kg higher than that at 35 °C, demonstrating that DMPP continuously inhibited ammonium-to-nitrate transformation.

Overall, the controlled-release and inhibitory effects of CYRF were influenced by temperature, water penetration, membrane diffusion, concentration gradients, DMPP release, pH, and nitrifying microbial activity. The coordination between urea nitrogen release and inhibitor action maintained ammonium nitrogen supply while restricting nitrate nitrogen formation, resulting in synergistic regulation through “controlled release–inhibited transformation.

### 3.5 Changes in soil nitrogen contents under different fertilizer treatments at the same temperature

#### 3.5.1 Characteristics of nitrogen content changes under different fertilizer treatments at the same temperature

**Figure 5:**
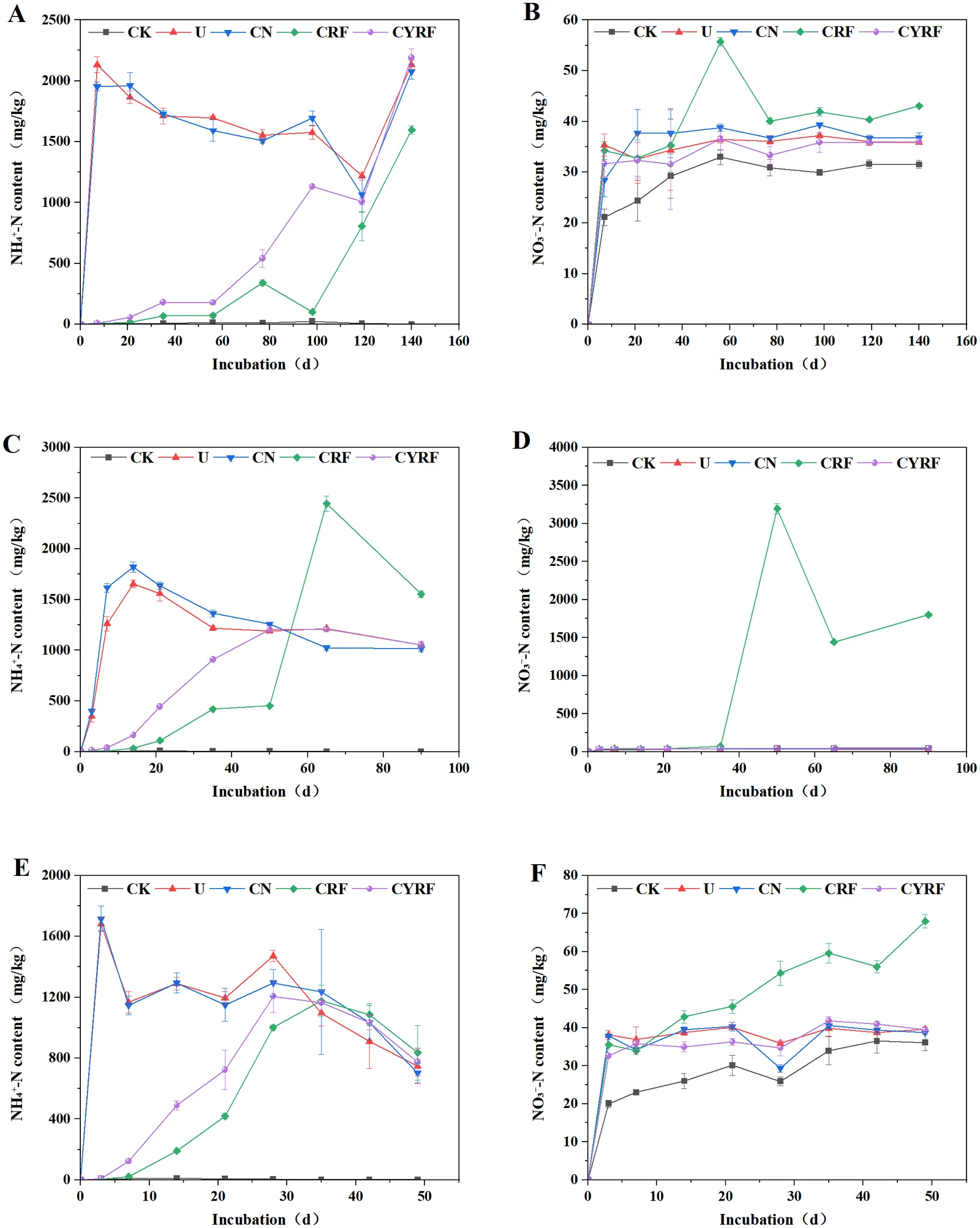
Soil inorganic nitrogen under different treatments and temperatures. **(A–F)** show soil ammonium and nitrate nitrogen at 15, 25, and 35 °C, respectively. CK, blank control; U, conventional urea; CN, stabilized fertilizer; CRF, controlled-release fertilizer; CYRF, controlled-release inhibitor-enhanced fertilizer.

To further identify differences in nitrogen supply timing and nitrogen transformation among fertilizer systems under the same thermal condition, soil ammonium nitrogen and nitrate nitrogen dynamics were compared among CK, U, CN, CRF, and CYRF treatments at each temperature (**Figure 5**).

At 15 °C, ammonium nitrogen levels differed markedly among treatments, while nitrate nitrogen accumulation remained limited. Low temperature generally slowed urea nitrogen release, ammonium nitrogen formation, and nitrification. The delayed early-stage increases under CRF and CYRF reflected the controlled-release effect of the coating. At 21, 35, and 90 d, CYRF increased ammonium nitrogen by 151.8%, 58.8%, and 37.3%, respectively, relative to CRF. These results indicate that CYRF more effectively suppressed nitrification and other ammonium-consuming processes, thereby maintaining ammonium nitrogen supply during the later incubation stage.

For nitrate nitrogen at 15 °C, CYRF showed decreases of 34.4% and 16.4% relative to CRF at 21 and 90 d, respectively. This indicates that although CRF delayed fertilizer nitrogen release, it had limited capacity to prevent the conversion of released ammonium nitrogen into nitrate nitrogen.

At 25 °C, U and CN rapidly formed ammonium nitrogen, whereas CRF showed delayed release. Compared with CRF, CYRF increased ammonium nitrogen at 21 and 50 d by 305.4% and 165.5%, respectively, but decreased it at 65 and 90 d by 50.6% and 32.0%, respectively. These results suggest that CYRF avoided excessive ammonium accumulation during the later stage and maintained a more stable nitrogen supply pattern.

At 25 °C, CYRF reduced nitrate nitrogen by 98.7%, 97.0%, and 97.6% relative to CRF at 50, 65, and 90 d, respectively. Although CRF delayed urea nitrogen release, CYRF additionally controlled ammonium-to-nitrate transformation after release, thereby maintaining a relatively stable ammonium nitrogen supply and substantially suppressing nitrate nitrogen accumulation.

At 35 °C, nitrogen release and transformation occurred earlier across all treatments. U and CN formed high levels of ammonium nitrogen at the early stage, but at 49 d they retained only 44.5% and 40.9% of their peak values, respectively, indicating that high temperature accelerated ammonium nitrogen depletion. The peak values of CRF and CYRF also appeared earlier than those at 25 °C, showing that increasing temperature accelerated urea nitrogen release from coated fertilizers. Nevertheless, at 49 d, CRF and CYRF still maintained relatively high ammonium nitrogen levels, indicating that the controlled-release structure could still prolong part of fertilizer nitrogen release into the middle and late stages.

For nitrate nitrogen at 35 °C, CRF showed a more pronounced increase during the later stage. At 49 d, nitrate nitrogen under CRF was approximately 1.89, 1.72, 1.75, and 1.72 times the corresponding values under CK, U, CN, and CYRF, respectively. At 49 d, nitrate nitrogen under CYRF was 28.57 mg kg^-^¹ lower than that under CRF, corresponding to a decrease of approximately 42.0%, indicating that the inhibitor component still effectively limited nitrate nitrogen accumulation. Overall, at 35 °C, elevated temperature shortened the interval between fertilizer nitrogen release and soil nitrogen transformation. Compared with CRF, CYRF better balanced ammonium nitrogen supply during the middle and late stages while controlling nitrate nitrogen accumulation.

### 3.6 Biomass reponses of winter wheat and summer maize to different fertilization treatments

**Figure 6:**
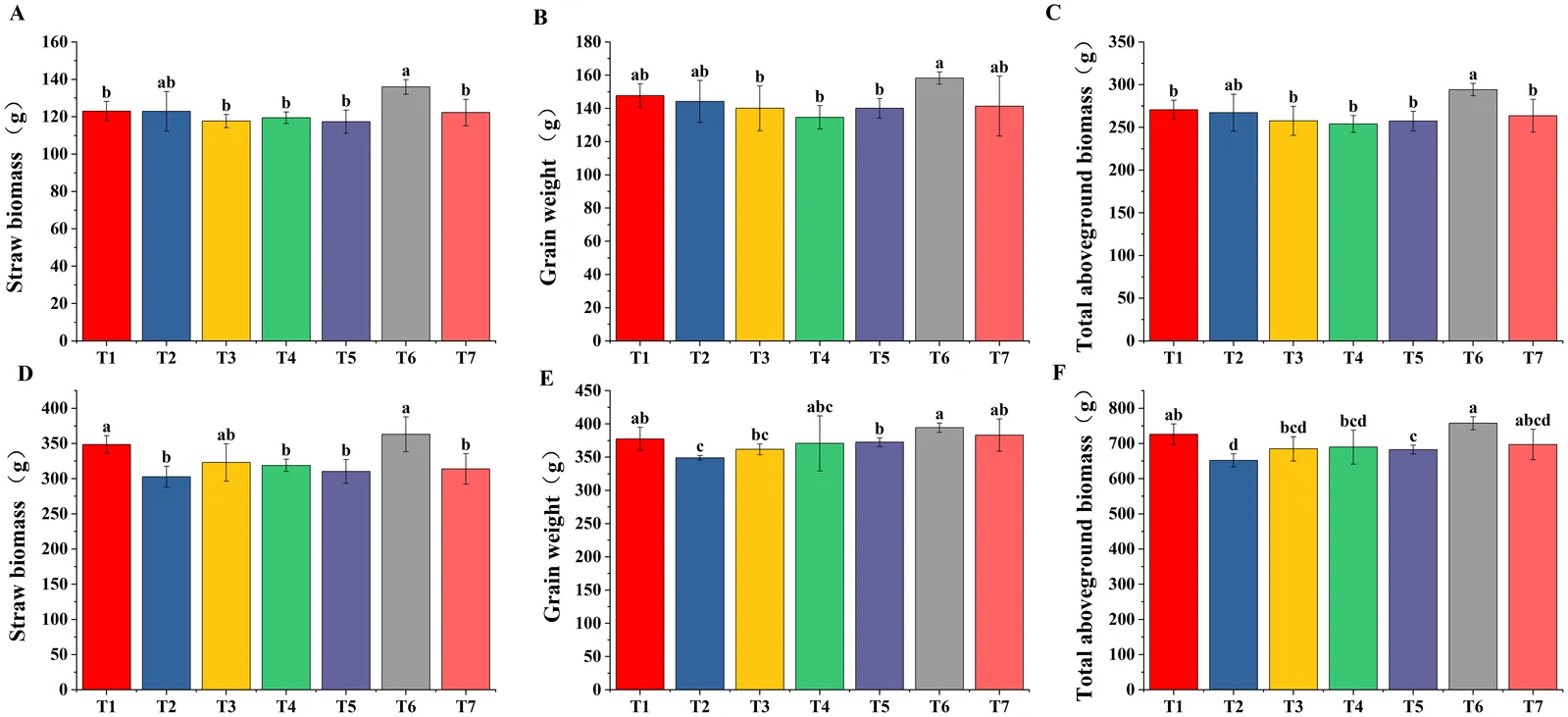
Biomass of winter wheat and summer maize under different treatments. **(A–C)**, winter wheat grain, straw, and above-ground biomass; **(D–F)**, summer maize straw, grain, and above-ground biomass. T1–T3, conventional urea; T4–T5, CRF; T6–T7, CYRF. Different letters indicate significant differences according to the LSD test at P < 0.05.

The biomass responses of winter wheat and summer maize to different fertilization treatments are shown in **Figure 6**. In winter wheat, the T6 treatment produced relatively high straw dry weight, grain dry weight, and total above-ground biomass, with values of 135.93 g, 158.33 g, and 294.26 g, respectively. Straw dry weight under T6 was 10.5%, 13.8%, and 15.8% higher than that under T1, T4, and T5, respectively. Straw dry weight under T6 was significantly higher than that under the other treatments. Grain dry weight under T6 was also 7.2%, 17.6%, and 13.0% higher than that under T1, T4, and T5, respectively, although these differences were not statistically significant. For total above-ground biomass, T6 was 8.7%, 15.8%, and 14.3% higher than T1, T4, and T5, respectively. T6 was significantly higher than T3, T4, T5, and T7, but not significantly different from T1 and T2.

For summer maize, the stem dry weight, grain dry weight, and total above-ground biomass under the T6 treatment were 363.05 g, 394.33 g, and 757.38 g, respectively. For stem dry weight, T6 was 4.2%, 13.9%, and 17.0% higher than T1, T4, and T5, respectively, and was significantly higher than the other treatments. For grain dry weight, T6 was 4.4%, 6.4%, and 5.8% higher than T1, T4, and T5, respectively, although the differences were not significant. For total above-ground biomass, T6 was 4.3%, 9.8%, and 10.9% higher than T1, T4, and T5, respectively. It was significantly higher than T2, T3, T4, T5, and T7.

Compared with the CRF treatments, the CYRF treatments were more favorable for above-ground dry matter accumulation, which may be associated with the synergistic effects of controlled release and inhibitor-mediated regulation of nitrogen transformation.

### 3.7 Changes in nitrogen utilization and loss rates under different fertilization treatments based on ¹^5^N tracing

To further clarify how CYRF affected the fate of fertilizer-derived nitrogen, ¹^5^N tracing was used to compare nitrogen utilization, residual nitrogen, and nitrogen loss rates among different fertilization treatments. Because the ¹^5^N-labeling strategies differed among treatments, comparisons were made only among treatments with the same labeling pattern. In this comparison, T4 and T6 represented the ¹^5^N fate of the target fertilizer components in the CRF and CYRF systems, respectively, whereas T5 and T7 represented the ¹^5^N fate of the complete CRF- and CYRF-based fertilization systems, respectively. The last column of **Tables 3** and **4** presents changes relative to the designated reference treatments, whereas the direct effects of CYRF relative to CRF were evaluated by comparing T6 with T4 and T7 with T5.

**Table 3:** ^15^N utilization, residual, and loss rates under different fertilization treatments in winter wheat.

Fertilizer treatment	^15^N utilization rate %	^15^N residual rate %	Loss rate %	Reduction in loss rate (percentage points)
T1	32.89±1.28 c	33.81±4.44 a	33.30	–
T4	52.42±3.11 b	27.50±3.35 ab	20.08	–
T6	67.02±0.55 a	24.35±2.55 b	8.63	11.45
T2	31.68±2.25 c	38.99±3.26 a	29.32	–
T3	37.71±4.66 c	32.48±2.50 b	29.81	-0.49
T5	46.77±0.28 b	36.87±1.74 a	16.36	12.96
T7	50.88±2.23 a	36.75±1.15 a	12.89	16.43

Values are means ± SD (n = 3). Different lowercase letters within the same column indicate significant differences among treatments with the same ^15^N-labeling pattern according to the LSD test at P < 0.05. Comparisons were made within the same ^15^N-labeling group: T2 was used as the reference for T3, T5, and T7, whereas T4 was used as the reference for T6. Negative values indicate an increase in the nitrogen loss rate.

**Table 4:** ^15^N utilization, residual, and loss rates under different fertilization treatments in summer maize.

Fertilizer treatment	^15^N utilization rate %	^15^N residual rate %	Loss rate %	Reduction in loss rate (percentage points)
T1	29.61±3.05 c	37.08±1.20 a	33.31	–
T4	41.10±3.41 b	31.62±1.99 b	27.28	–
T6	46.40±1.58 a	37.44±1.42 a	16.17	11.11
T2	27.77±1.01 d	32.80±3.61 b	39.43	–
T3	31.94±2.20 c	37.05±1.32 ab	31.01	8.42
T5	38.54±0.93 a	38.46±2.16 ab	23.00	16.43
T7	41.58±2.51 a	38.63±1.85 a	19.79	19.64

Values are means ± SD (n = 3). Different lowercase letters within the same column indicate significant differences among treatments with the same ^15^N-labeling pattern according to the LSD test at P < 0.05. Comparisons were made within the same ^15^N-labeling group: T2 was used as the reference for T3, T5, and T7, whereas T4 was used as the reference for T6.

In winter wheat, compared with T4, T6 increased the ¹^5^N utilization rate by 14.60 percentage points, and the difference was significant. The ¹^5^N residual rate decreased by 3.15 percentage points, but the difference was not significant, while the nitrogen loss rate decreased by 11.45 percentage points. Compared with T5, T7 increased the ¹^5^N utilization rate by 4.11 percentage points, and the difference was significant. The ¹^5^N residual rate decreased by 0.12 percentage points, with no significant difference, while the nitrogen loss rate decreased by 3.47 percentage points (**Table 3**).

In summer maize, compared with T4, T6 increased the ¹^5^N utilization and residual rates by 5.30 and 5.82 percentage points, respectively, and both differences were significant, while the nitrogen loss rate decreased by 11.11 percentage points. Compared with T5, T7 increased the ¹^5^N utilization rate by 3.04 percentage points and the residual rate by 0.17 percentage points, although neither difference was significant, while the nitrogen loss rate decreased by 3.21 percentage points (**Table 4**).

Overall, compared with CRF, the core fertilizer component of CYRF achieved higher ¹^5^N utilization and lower nitrogen loss. At the whole fertilization-system level, CYRF also tended to enhance fertilizer-derived nitrogen utilization while reducing nitrogen loss.

## 4 Discussion

The performance of a controlled-release inhibitor-enhanced nitrogen fertilizer depends on two key factors: stable loading of the inhibitor onto urea granules and its subsequent dissolution and diffusion through the outer coating. Aqueous loading systems are sensitive to membrane water resistance, crosslinking, and drying conditions, whereas poor process control may cause losses of urea and active components. Organic solvents can reduce urea surface dissolution but involve volatilization, environmental, and safety concerns (Liu et al., 2024; Dovzhenko et al., 2024). Direct evidence that inhibitors can diffuse effectively through the coating after drying remains limited.

In this study, CYRF was prepared with an inner DMPP-containing layer and an outer controlled-release membrane, thereby combining regulation of urea nitrogen release with nitrification inhibition. A polyamino-acid-based material was used to dissolve, disperse, and load the inhibitor. SEM and EDS analyses confirmed a distinct composite coating with a porous structure. During static water incubation at 25 °C, CYRF showed no early burst release, and DMPP and urea nitrogen were released continuously with a strong positive correlation between their cumulative release rates (r = 0.975, P < 0.001). This indicates good synchronization between inhibitor release and nitrogen supply. Coated-fertilizer release is generally controlled by water penetration, core dissolution, solute diffusion, and membrane resistance, while carrier protection may prolong DMPP effectiveness (Sun et al., 2025; Wang et al., 2025).

DMPP effectiveness is also influenced by soil properties and environmental conditions (Guan et al., 2024). In the soil incubation experiment, CYRF enhanced ammonium nitrogen retention and restricted nitrate nitrogen accumulation across temperatures. In contrast, nitrogen transformation under U and CN was more temperature-sensitive. At 25 °C, nitrate nitrogen accumulated markedly during the middle and late stages under CRF, whereas CYRF showed no sharp increase at any temperature. Thus, CYRF regulated both nitrogen release and post-release transformation. Similar effects of DMPP on nitrification, crop nitrogen uptake, and nitrogen use efficiency in calcareous soil have recently been reported (Liu et al., 2026).

Previous studies have shown that placing inhibitors between the fertilizer core and outer membrane can improve nitrogen use efficiency, although evaluations have mainly relied on yield, apparent nitrogen use efficiency, and soil inorganic nitrogen (Sun et al., 2025). Here, ¹^5^N tracing showed that T6 increased crop recovery of fertilizer-derived nitrogen and reduced nitrogen loss relative to T4. The comparison between T7 and T5 further indicated that this advantage remained within the integrated fertilization system. CYRF therefore improved the match between fertilizer nitrogen supply and crop nitrogen demand, distinguishing it from conventional controlled-release fertilizers.

Overall, CYRF combined controlled nitrogen release with nitrification inhibition and showed potential for one-time and reduced-nitrogen fertilization. However, NH_3_ volatilization, N_2_O emissions, deep-soil nitrate leaching, and nitrification-related functional genes were not measured. Future studies should integrate gaseous nitrogen losses, deep-soil nitrogen migration, crop nitrogen uptake, and microbial indicators to further clarify its mechanisms.

## 5 Conclusion

This study reveals the intrinsic mechanism by which the polyurethane controlled-release membrane coupled with a DMPP nitrification inhibitor achieves the coordinated release and transformation of urea nitrogen. Membrane structure analysis confirmed that the outer polyurethane coating and inner DMPP layer established a “slow-release first, inhibition second” configuration. Release kinetics demonstrated high synchrony between DMPP and urea nitrogen, ensuring their near-simultaneous arrival at the soil interface and providing the spatiotemporal prerequisite for effective nitrification inhibition. Soil incubation further showed that, compared with controlled-release fertilizer alone, this coupled regulation consistently enhanced ammonium retention at low to moderate temperatures and significantly suppressed nitrate accumulation at high temperatures, reflecting effective protection and sustained inhibitor supply by the membrane. Field ¹^5^N tracing validated the agronomic benefits—improved nitrogen use efficiency in winter wheat and reduced apparent nitrogen loss during both the winter wheat and summer maize growing seasons—confirming that this approach was environmentally favorable without compromising yield. By modulating release kinetics via membrane structure, a temporal window was created for the inhibitor to exert its targeted function in the rhizosphere. This finding offers a theoretical basis for overcoming the limitations of single-technology fertilizers and provides insights for the structural design and application of next-generation high-efficiency fertilizers.

## Data Availability

The raw data supporting the conclusions of this article will be made available by the authors, without undue reservation.
