# Correction: Preparation, characterization, and performance evaluation of a controlled-release inhibitor-enhanced nitrogen fertilizer

**DOI:** 10.3389/fpls.2026.1963941

**Published:** 2026-09-02

**Authors:** Frontiers Production Office

**Keywords:** ^15^N tracing, 3,4-dimethylpyrazole phosphate (DMPP), controlled-release inhibitor-enhanced nitrogen fertilizer, nitrification inhibition, nitrogen use efficiency

The contents of **Table 3** were duplicated and replaced the contents of **Table 4**. The original content of **Table 4** has been reinstated.

**Table 3:** ^15^N utilization, residual, and loss rates under different fertilization treatments in winter wheat.

Fertilizer treatment	^15^N utilization rate %	^15^N residual rate %	Loss rate %	Reduction in loss rate (percentage points)
T1	32.89±1.28 c	33.81±4.44 a	33.30	–
T4	52.42±3.11 b	27.50±3.35 ab	20.08	–
T6	67.02±0.55 a	24.35±2.55 b	8.63	11.45
T2	31.68±2.25 c	38.99±3.26 a	29.32	–
T3	37.71±4.66 c	32.48±2.50 b	29.81	-0.49
T5	46.77±0.28 b	36.87±1.74 a	16.36	12.96
T7	50.88±2.23 a	36.75±1.15 a	12.89	16.43

Values are means ± SD (n = 3). Different lowercase letters within the same column indicate significant differences among treatments with the same ^15^N-labeling pattern according to the LSD test at P < 0.05. Comparisons were made within the same ^15^N-labeling group: T2 was used as the reference for T3, T5, and T7, whereas T4 was used as the reference for T6. Negative values indicate an increase in the nitrogen loss rate.

**Table 4:** ^15^N utilization, residual, and loss rates under different fertilization treatments in summer maize.

Fertilizer treatment	^15^N utilization rate %	^15^N residual rate %	Loss rate %	Reduction in loss rate (percentage points)
T1	29.61±3.05 c	37.08±1.20 a	33.31	–
T4	41.10±3.41 b	31.62±1.99 b	27.28	–
T6	46.40±1.58 a	37.44±1.42 a	16.17	11.11
T2	27.77±1.01 d	32.80±3.61 b	39.43	–
T3	31.94±2.20 c	37.05±1.32 ab	31.01	8.42
T5	38.54±0.93 a	38.46±2.16 ab	23.00	16.43
T7	41.58±2.51 a	38.63±1.85 a	19.79	19.64

Values are means ± SD (n = 3). Different lowercase letters within the same column indicate significant differences among treatments with the same ^15^N-labeling pattern according to the LSD test at P < 0.05. Comparisons were made within the same ^15^N-labeling group: T2 was used as the reference for T3, T5, and T7, whereas T4 was used as the reference for T6.

The original version of this article has been updated.

